# Diagnosis and Treatment of Low-Grade Marginal Zone B-cell Lymphoma With Psychiatric Overlap

**DOI:** 10.7759/cureus.59735

**Published:** 2024-05-06

**Authors:** Abraham A Mascio, Devaun M Reid, Britannia O Noel, Dwight Smith Jr., Martin Giangreco

**Affiliations:** 1 Internal Medicine, University of South Florida Morsani College of Medicine, Tampa, USA; 2 College of Medicine, University of South Florida Morsani College of Medicine, Tampa, USA

**Keywords:** marginal zone b-cell lymphoma, catatonia, neurocognitive impairment, clinical psychiatry, bipolar

## Abstract

This case report delineates the intricate interplay between psychiatric and oncological pathology in a 72-year-old male diagnosed with low-grade marginal zone B-cell lymphoma and severe psychiatric disturbances, including catatonia. The presentation of severe psychiatric symptoms initially obscured the underlying lymphoma, delaying diagnosis and complicating clinical management. Notably, the lymphoma itself may have precipitated or exacerbated the psychiatric condition, underscoring the potential for oncological diseases to manifest with rapidly progressive dementia and catatonia. A multidisciplinary approach was employed, utilizing electroconvulsive therapy (ECT) for rapid resolution of catatonia, which facilitated significant mental health improvements and clearer delineation of the oncological underpinnings. Concurrently, the patient was treated with rituximab, targeting the lymphoma. This case highlights the critical need for a comprehensive evaluation in patients presenting with psychiatric symptoms, particularly in the elderly, to uncover potential medical causes and illustrates the efficacy of ECT in managing psychiatric conditions that may overshadow or complicate concurrent medical issues.

## Introduction

This case report investigates the confluence of low-grade marginal zone B-cell lymphoma, a type of slowly progressing non-Hodgkin lymphoma, and catatonia, a severe psychiatric syndrome characterized by motor and behavioral disturbances such as mutism, stupor, or extreme agitation. Instances where central nervous system (CNS) involvement by this lymphoma leads to neurological symptoms have been documented, illustrating a complex overlap of psychiatric and medical issues [1,2]. This report presents a 74-year-old male with a history of bipolar disorder and recent cognitive decline, where the clinical presentation blurred the lines between psychiatric manifestations and medical pathology. Additionally, this case highlights the effectiveness of electroconvulsive therapy (ECT) in treating catatonia, which significantly improved the clinical outcome and enhanced the overall management of his lymphoma.

## Case presentation

**Figure 1:**
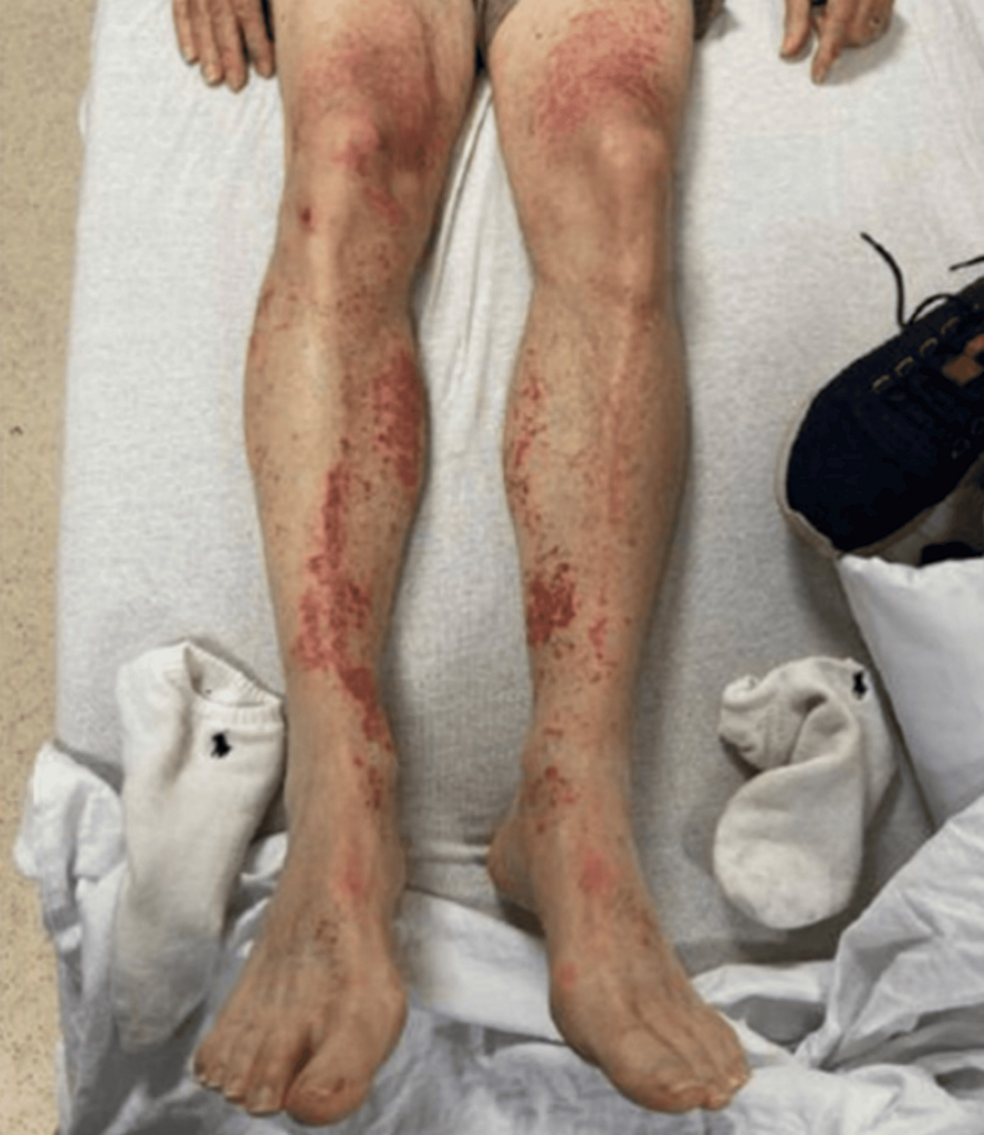
Diffuse seborrheic dermatitis on the posterior torso. Bilateral lower extremity non-blanching macular rash on anterior thigh and anterior shin

We present the case of a 72-year-old white male with a history of neurocognitive disorder, bipolar disorder with catatonia, and lymphoproliferative disorder to the emergency room (ER) with worsening mental status and catatonia, speech difficulties, and diffuse rashes. The patient's vital signs were recorded as follows: the temperature was 97.5°F (36.4°C), pulse rate was 90 bpm, respiratory rate was 18 breaths per minute, blood pressure was 131/76 mmHg, and oxygen saturation (SpO2) was 98% on room air. In light of the patient's complex medical history and the presentation of his symptoms, the differential diagnosis for the rash primarily included vasculitis, capillaritis, and possible exacerbation of preexisting autoimmune or inflammatory disorders. Considering these possibilities, the physicians commenced symptomatic treatment of the rash and initiated a therapeutic trial of prednisone to address potential inflammatory etiologies (Figure 1).

**Figure 2:**
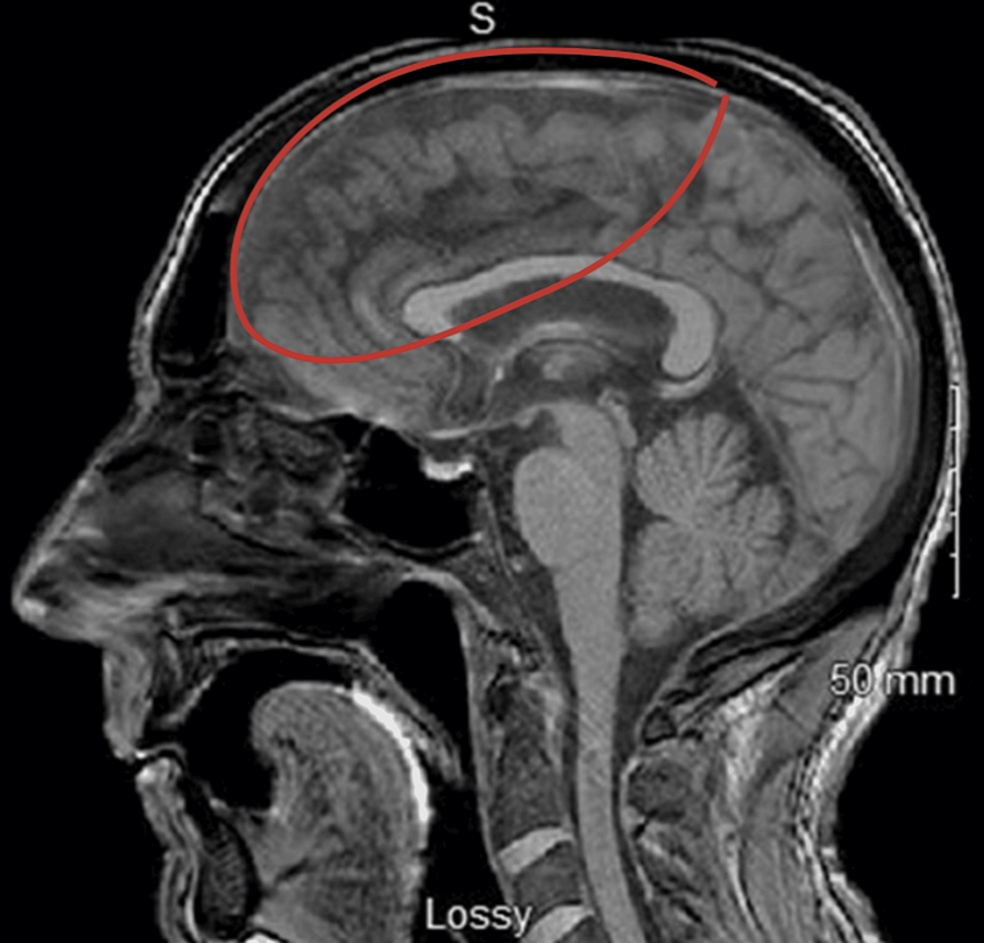
Mild generalized nonspecific pachymeningeal thickening throughout the cranium due to uncertain etiology

After being treated with prednisone, the patient was discharged. Four days later, he and his wife returned to the ER due to episodes of altered mental status (AMS) and mania. Following a psychiatric consultation, the patient was prescribed olanzapine to address his manic episodes, which effectively mitigated these symptoms but resulted in notable confusion, loss of concentration, loss of environmental awareness, and speech difficulties. As additional complications like insomnia, potential depression, and a significant loss of appetite emerged, 45 mg mirtazapine at night was introduced to his regimen to address these symptoms. Due to the development of akathisia, a rapid switch from 2.5 mg olanzapine twice daily to 50 mg sertraline daily was implemented. However, one week later, the patient exhibited increasingly severe catatonic symptoms, including delayed and reduced speech, restlessness, withdrawal from social interactions, and aggressive behaviors toward his wife, which led to his hospital admission. These psychiatric presentations initially managed as primary psychiatric disorders, may have been exacerbated or directly influenced by an underlying lymphoma. In the hospital, the consulting team recommended ECT and obtained further diagnostic imaging. This imaging revealed mild parenchymal inflammation and a left adrenal mass (Figure 2).

At this stage, the patient experienced hypoxia due to complications from a previous COVID-19 infection, necessitating intubation. Sedating medications used for endotracheal intubation and mechanical ventilation can impair cognitive function by depressing CNS activity, potentially exacerbating cognitive deficits in this patient. It took several days for him to stabilize, during which he showed fluctuating catatonic symptoms. Following ECT, a lumbar puncture was performed, and flow cytometry analysis of the cerebrospinal fluid (CSF) identified a significant B-cell clonal population with the CD19 positive, CD20 positive, CD5 negative, CD10 negative, and surface Kappa light chain positive immunophenotype. The patient was then scheduled for a bone marrow biopsy, which confirmed low-grade marginal zone B-cell lymphoma, showing clonal populations of B-cells. Following consultation, the patient was initiated on four doses of intravenous rituximab, scheduled for weekly infusions over a period of four weeks, with consideration for future intrathecal rituximab administration. He received the first two doses during his inpatient stay. The patient was started on rituximab mono-therapy, primarily due to its efficacy in targeting B-cell lymphomas with fewer side effects. The patient's condition improved following the initiation of ECT for catatonia and intravenous rituximab treatment for marginal zone B-cell lymphoma, with plans for ongoing management by oncology, psychiatry, and primary care.

## Discussion

This case report underscores the diagnostic complexity faced when treating a 72-year-old male with an intricate interplay of psychiatric symptoms and newly diagnosed low-grade marginal zone B-cell lymphoma. His clinical scenario was further complicated by the simultaneous presence of severe psychiatric disorders, including catatonia, neurocognitive impairments, and hematologic malignancy, both influencing each other’s clinical presentations and therapeutic responses.

The diagnostic process was notably challenged by the patient’s extensive polypharmacy regimen. The myriad medications introduced a significant complexity to his management, as the side effects of each drug could mimic or obscure the manifestations of his primary medical conditions. This required meticulous medication management and adjustments to ensure that symptoms were not being masked by drug effects, underscoring the necessity for thorough medication reviews in similar complex cases [3-5].

Early diagnosis in this case could have potentially been facilitated by heightened awareness and screening for organic causes of psychiatric symptoms, particularly in elderly patients presenting with new or significantly altered mental health symptoms. Earlier integration of neuroimaging, more frequent psychiatric evaluations, and perhaps earlier consideration for bone marrow biopsy upon initial presentation of unexplained neurocognitive changes might have led to a quicker understanding of the underlying lymphoma [6,7].

ECT uses electrical currents to induce seizures for therapeutic effects. It employs either a sinusoidal wave or brief-pulse ECT, with the latter being more common due to its efficacy and fewer cognitive side effects. The ECT dose is calculated based on the individual's seizure threshold, determined by initial titration sessions, and is typically set at 1.5 to 2.5 times this threshold to balance efficacy and minimize side effects. The ability of ECT to provide rapid symptom relief was indispensable in delineating between psychiatric and organic causes of catatonia, thereby guiding more targeted medical and psychiatric care [8-10]. Furthermore, the diagnosis of marginal zone B-cell lymphoma highlighted through CD19 and CD20 positivity in the bone marrow exemplifies the need for a multidisciplinary treatment approach. This approach was crucial, as the lymphoma’s potential CNS involvement could mimic or intensify neurological and psychiatric symptoms, necessitating the concurrent management of oncologic and psychiatric conditions. The employment of rituximab, a CD20 targeting agent, was pivotal in managing the lymphoma effectively, demonstrating the importance of personalized and precise therapy in managing overlapping medical and psychiatric conditions.

## Conclusions

This case illustrates the paramount importance of considering a holistic and multidisciplinary approach in patients with complex presentations. It emphasizes the need for early consideration of organic causes in the differential diagnosis of psychiatric conditions, particularly in patients with atypical presentations or those resistant to standard psychiatric treatments. The integration of ECT in managing catatonia, whether primary or secondary to another medical condition, showcases its role in complex cases where traditional psychiatric interventions may prove inadequate. This approach ensures comprehensive management of all underlying causes, thereby enhancing patient outcomes amid diagnostic and therapeutic challenges.
